# Application of Exogenous dsRNAs-induced RNAi in Agriculture: Challenges and Triumphs

**DOI:** 10.3389/fpls.2020.00946

**Published:** 2020-06-25

**Authors:** Protiva Rani Das, Sherif M. Sherif

**Affiliations:** Alson H. Smith Jr. Agricultural Research and Extension Center, School of Plant and Environmental Sciences, Virginia Polytechnic Institute and State University, Winchester, VA, United States

**Keywords:** exogenous dsRNAs, plant RNAi, application method, delivery technique, symplastic movement, apoplastic movement, agricultural traits

## Abstract

In recent years, RNA interference (RNAi) machinery has widely been explored by plant biologists for its potential applications in disease management, plant development, and germplasm improvement. RNAi-based technologies have mainly been applied in the form of transgenic plant generation and host-induced-gene-silencing (HIGS). However, the approval of RNAi-based transgenic plants has always been challenging due to the proclaimed concerns surrounding their impacts on human health and the environment. Lately, exogenous applications of double-stranded RNAs (dsRNAs), short interfering RNAs (siRNAs), and hairpin RNAs (hpRNAs) has emerged as another technology that could be regarded as more eco-friendly, sustainable, and publicly acceptable than genetic transformation. Inside the plant cell, dsRNAs can undergo several steps of processing, which not only triggers RNAi machinery but may also involve transitive and systemic silencing, as well as epigenetic modifications. Therefore, along with the considerations of proper exogenous applications of dsRNAs, defining their final destination into plant cells is highly relevant. In this review, we highlighted the significance of several factors that affect dsRNA-induced gene silencing, the fate of exogenous dsRNAs in the plant cell, and the challenges surrounding production technologies, cost-effectiveness, and dsRNAs stability under open-field conditions. This review also provided insights into the potential applications of exogenous dsRNAs in plant protection and crop improvement.

## Introduction

RNA interference (RNAi) is a natural gene silencing phenomenon and recently is being extensively used in agriculture to improve traits related to disease management, plant development, and crop improvement. Applications of RNAi technology in agriculture are largely based on transgenic approaches, where transgenic plants express double-stranded RNAs (dsRNAs) to silence specific genes that control target traits (Qi et al., 2019). However, the development and maintenance of transgenic plants are costly and not yet technologically achievable for most horticultural crops (Andow and Zwahlen, 2006; Dalakouras et al., 2020). Furthermore, genetically modified (GM) plants have always been surrounded by public debates concerning their potential consequences on ecological systems and human health. From these perspectives, exogenously applied dsRNAs to induce gene silencing have been perceived as another alternative to the genetic transformation that could provide similar benefits, without risking ecological stability and societal acceptance (Dubrovina and Kiselev, 2019; Dalakouras et al., 2020). Indeed, several studies have reported that induction of RNAi mechanism by exogenous dsRNAs, short interfering RNAs (siRNAs), or hairpin RNAs (hpRNAs) has the potential to protect plants against plant pathogenic viruses (Tenllado and Diaz‐Ruiz, 2001; Carbonell et al., 2008; Yin et al., 2009; Gan et al., 2010; Konakalla et al., 2016; Vadlamudi et al., 2020), fungi (Koch et al., 2016; Wang et al., 2016; Wang et al., 2017), insects (Baum et al., 2007; Li et al., 2013; Ghosh et al., 2017; Luo et al., 2017), mites, and nematodes (reviewed in Dubrovina and Kiselev, 2019; Dalakouras et al., 2020), which could eventually reduce the ecological footprints caused by chemical pesticides. However, it should be noted that most of the studies on the efficacy of exogenously applied dsRNAs were carried out under set experimental conditions, e.g., using detached leaves, targeting of transgenes, co-inoculation of dsRNAs with target viruses, etc, and have rarely been implemented under open-field conditions where several factors can largely affect their stability, uptake, and overall applicability.

Several factors could affect the efficiency of exogenously applied dsRNA-induced RNAi in plants including, but are not necessarily limited to, concentration/dose and length/size of dsRNAs, application method, delivery technique, plant organ-specific activities, and stability under unseemly environmental conditions. These factors eventually determine the absorption/uptake rate of exogenous dsRNAs by plant cells to trigger RNAi. Inside the plant cell, dsRNAs are processed into siRNAs and follow several other steps before triggering the RNAi machinery (Meister and Tuschl, 2004). The fate of dsRNAs in the plant may also involve other mechanisms including symplastic and apoplastic movements, local and systemic silencing, as well as, DNA methylation and histone modifications (Dalakouras et al., 2020; Wang and Dean, 2020). However, the complete mechanisms by which the exogenously applied dsRNAs induce plant RNAi is still far from being clear. The main objective of this review is to present an overview of possible factors that might affect RNAi induction by exogenously applied dsRNAs, and to discuss the role of exogenous dsRNAs in plant RNAi regulation and their potential fate into plant cells.

### Induction of Plant RNAi by dsRNAs

In plants, RNAi is a gene silencing phenomenon that involves sequence-specific suppression of genes, which can be induced by dsRNAs precursor that may vary in length and origin (Waterhouse et al., 2001). In plant cellular system, dsRNAs are mainly processed into three categories: short interfering RNAs (siRNAs), micro RNAs (miRNAs), and piwi interacting RNAs (piRNAs). Generally, siRNAs and miRNAs are collectively known as small RNAs (sRNAs) (Carthew and Sontheimer, 2009). The possible cellular mechanism of dsRNA induced RNAi in plants involves the following steps (Meister and Tuschl, 2004; Dalakouras et al., 2020): (i) Upon cellular uptake of dsRNAs, DICER-LIKE (DCL) endonucleases rapidly cleavages them into 20 to 25-nucleotide siRNAs with 2-nt 3´ overhangs at both ends; (ii) One strand of siRNAs is incorporated into an ARGONAUTE (AGO) protein to form an RNA-induced silencing complex (RISC); (iii) Finally, the siRNA molecules guide the RISC to scan the cytoplasm for recognition and cleavage/degradation of the complementary transcripts, thus resulting in post-transcriptional gene silencing (PTGS).

### Considerable Factors For Exogenous dsRNAs Application

**Figure 1:**
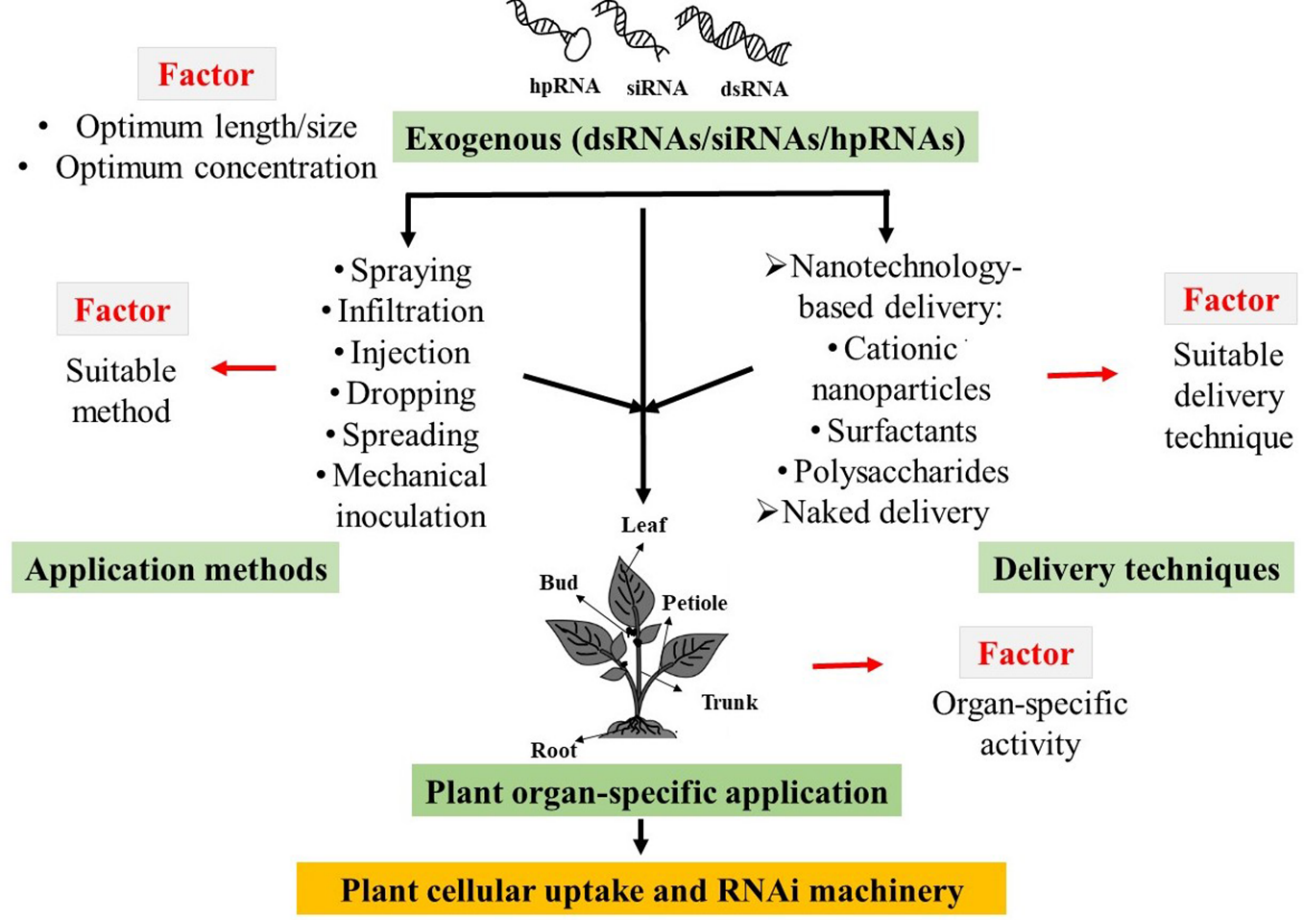
Schematic diagram of possible factors that influences the exogenous double-stranded RNAs (dsRNAs), short interfering RNAs (siRNAs), and hairpin RNAs (hpRNAs) induced RNA interference (RANi) in plant.

Successful cellular uptake and subsequent initiation of target gene silencing by exogenous dsRNAs are subject to the influences of length and/or concentrations, application methods, and delivery techniques, as well as the sensitivity of plant organs to dsRNA applications (Numata et al., 2014; Dalakouras et al., 2016; Mitter et al., 2017; Dubrovina and Kiselev, 2019). To date, very little is known regarding how these factors influence the exogenous dsRNAs-induced gene silencing in plant systems. Therefore, it is necessary to find out the optimal physical conditions along with the exogenous application method of external dsRNAs to develop eco-friendly approaches for plant protection and improvement of crop productivity (**Figure 1**).

In general, exogenous application methods include spraying, infiltration, injection, spreading, mechanical inoculation, and root/seed soaking, and they all have been widely used to apply dsRNAs/siRNAs/hpRNAs onto plants for target gene silencing (**Table 1**). When high-pressure spraying was used for the exogenous application of siRNAs, it was successful in inducing local and systemic silencing of the green fluorescent protein (*GFP*) transgene in *Nicotiana benthamiana* (Dalakouras et al., 2016). According to this study, high-pressure spraying was more efficient compared to wiping, infiltration, and gene gun methods. In contrast, another study reported that direct exogenous application of dsRNA by spreading without using any additional techniques induced efficient suppression of enhanced green fluorescent protein (*EGFP*) and neomycin phosphotransferase–II (*NPTII*) transgenes in *Arabidopsis* (Dubrovina et al., 2019). The authors of this study also analyzed the effects of different dsRNA concentrations (0.1, 0.35 and 1.0 µg/µl) and the results indicated that optimum concentration (0.35 µg/µl) had a higher significant influence on transgene-silencing efficiency (Dubrovina et al., 2019). The effects of different lengths of dsRNAs (315, 596, and 977-bp) targeting different virus genes were also investigated in *N. tabacum* leaves and results indicated that shorter dsRNAs showed reduced antiviral activity, indicating that dsRNA length could influence on its efficacy (Tenllado and Diaz‐Ruiz, 2001).

**Table 1:** Application methods, delivery techniques, length/concentrations, and organ-specific applications of exogenous dsRNAs in plants.

Type of RNAi molecules	Application methods	Delivery techniques	dsRNA length/size	dsRNA concentration/dose	Plant organ-specific application	Goal/target of RNAi	Detection of siRNAs	Reference
dsRNAs	Infiltration	Peptide complex-based delivery, Naked delivery		100 µl (20 pmol)	Mature leaves of *Arabidopsis thaliana* and poplar (*Populus tremula × tremuloides*)	Down-regulated target *YEP* transgene in *A. thaliana* and poplar, Local suppression of anthocyanin biosynthesis in *A. thaliana*		Numata et al., 2014
dsRNAs	Spraying	Layered double hydroxide (LDH) clay nanosheets-based delivery, Naked delivery	977 bp for *PMMoVIR54* and 330 bp for *CMV2b* viral gene, 504 bp for *GUS* gene	100 µg	Seeds of *A. thaliana,* Leaves of *Vigna unguiculate* and *Nicotiana tabacum*	Down-regulated target *GUS* transgene in *A.* seedling, Reduced local lesions numbers caused by CMV and PMMoV virus in *V. unguiculate* and *N. tabacum*	vsiRNAs from *N. tabacum* tissue samples with and without CMV inoculation using small-RNA sequencing	Mitter et al., 2017
dsRNAs	Root soaking	Cationic fluorescent nanoparticle G2-based delivery, Naked delivery	450 bp for *STM* gene, 550 bp for *WER* gene	1 µg	Roots of *Arabidopsis*	Down-regulated target *STM and WER* endogenous gene		Jiang et al., 2014
dsRNAs	Spreading	Naked delivery	720 bp for *EGFP* gene, 599 bp for *NPTII* gene	0.1 µg/µl, 0.35 µg/µl, and 1 µg/µl (100 µl/plant)	Surface of *A. thaliana*	Down-regulated target *EGFP* and *NPTII* transgene	*EGFP*-derived siRNA by stem-loop RT-PCR	Dubrovina et al., 2019
dsRNAs	Root soaking	Naked delivery	554 bp for *Mob1A* gene, 562 bp for *WRKY23* gene	1.0 mg/ml (1 ml for seedling soaking)	Seed of *A. thaliana*, rice, and maize	Down-regulated target *Mob1A* and *WRKY23* endogenous gene in *A. thaliana*		Li et al., 2015
siRNAs	High-pressure spraying	Naked-delivery	21-, 22-, and 24- nt siRNAs	100 µl of aqueous siRNA solutions (10 µM)	Leaves of *N.* *benthamiana*	Local and systemic silencing of the *GFP* transgene	Small RNA deep sequencing	Dalakouras et al., 2016
hpRNA, siRNAs	Spraying, Petiole absorption, Trunk injection	Naked- delivery	500-nt hpRNA, 21-, 22-, and 24-nt siRNAs	Trunk injection: hpRNA (1 ml, 500 µg), Petiole absorption: hpRNA (200 µl, 50 µg), Spraying: siRNA (8 µM)	*Vitis vinifera* and *N. benthamiana*	Local and systemic silencing	*GFP*- siRNAs by Northern blots	Dalakouras et al., 2018
dsRNA	Mechanical inoculation	Bacterial expression-based delivery	430 bp for *DhMYB1* gene	50 µl (2 µg/µl)	Flower bud of *Dendrobium hybrid*	Down-regulated target *DhMYB1* endogenous gene		Lau et al., 2015
dsRNAs	Co-inoculation with target virus	Naked delivery	315, 596, and 977 bp for PMMoV, 1483 bp for TEV, 1124 bp for AMV	5 µl (2.5 µM)	Leaf of *N. tabacum*	Virus resistance		Tenllado and Diaz‐Ruiz, 2001
dsRNA	Co-inoculation with target virus	Naked delivery	Viroid-specific dsRNAs	1250 and 5000 molar excess	Young leaves of: *Lycopersicon esculentum*, *Gynura aurantiaca*, and *Dendranthema grandiflora*	Virus resistance		Carbonell et al., 2008
dsRNAs	Co-inoculation with virus using spraying and mechanical rubbing	Bacterial expression-based delivery, Naked delivery		5 µg	Leaf of *N. benthamiana*	Virus resistance		Niehl et al., 2018
dsRNAs	Co-inoculation with target virus	Naked-delivery	588 bp for ZYMV *HC-Pro* gene, 498 bp for ZYMV *CP* gene	20 µl (40–60 µg)	Leaf of three cucurbit species: *Citrulus lanatus, Cucurbita pepo,* and *Cucumis sativus*	Virus resistance	ZYMV-derived vsiRNAs (dsRNA *HC-Pro*) by stem-loop RT-PCR in local and systemic leaves	Kaldis et al., 2018
dsRNA, sRNAs derived from DICER-cleaved dsRNA	Spraying	Naked-delivery	791-nt for *CYP3* genes of *Fusarium graminearum*	10 µg of dsRNA and siRNAs	Detached leaf and seedlings of *Hordeum vulgare*	Fungal resistance	*CYP3*-dsRNA-derived siRNA by Northern blots in local and distal (semi-systemic) leaf areas	Koch et al., 2016
dsRNA, sRNAs	Dropping onto the surface of each plant specimen	Naked delivery	252 bp for *DCL1* gene, 238 bp for *DCL2* gene, and 315 bp for *DCL1/2* (164 bp of *DCL1* and 151 bp of *DCL2*) of *Botrytis cinerea* 156 bp for *DCL1* and 156 bp for *DCL2* of *Verticillium dahliae*	20 μl of dsRNA and sRNAs (20 ng/μl)	Fruits: *Solanum lycopersicum*, *Fragaria × ananassa*, and *Vitis labrusca* Vegetables: *Lactuca sativa* and *Allium cepa*, Flower petals: *Rosa hybrida* Transgenic *Arabidopsis* and *N. benthamiana* plant	Fungal resistance		Wang et al., 2016
dsRNA	Foliar application	Naked-delivery	250–500 bp targeting *B. cinereal* genes	10–20 μl	Leaves of *Brassica napus* and *Arabidopsis*	Fungal resistance		McLoughlin et al., 2018
dsRNA	Spraying	Naked-delivery		30–40 ng/μl	Leaves surface of: *C. sativus, Glycine max, Hordeum vulgare,* and *Triticum aestivum*	Fungal resistance		Gu et al., 2019

STM, shoot meristemless; WER, werewolf; EGFP, enhanced green fluorescent protein; NPTII, neomycin phosphotransferase-II; Mob1A, MOB kinase activator 1A; PMMoV, pepper mild mottle virus; CMV, cucumber mosaic virus; TEV, tobacco etch virus; AMV, alfalfa mosaic virus; ZYMV HC-Pro, zucchini yellow mosaic virus the helper component-proteinase; ZYMV cp, zucchini yellow mosaic virus coat protein; CYP3, cytochrome P450, family 3; DCL1, Dicer-like 1; DCL2, Dicer-like 2; DCL1/2, Dicer-like 1/2.

Plant cell contains complex cellular structures, such as the rigid cell wall that acts as a physical barrier to provide tensile strength and protection against several stresses (Islam et al., 2019). Therefore, the delivery of exogenous dsRNAs/siRNAs/hpRNAs into plant cells is considered the most crucial step in initiating RNAi machinery. Nanotechnology-based delivery and surfactants-based delivery methods were extensively used along with naked-dsRNAs application in plants (reviewed in Dubrovina and Kiselev, 2019; Dalakouras et al., 2020). The major limitation of exogenous applications of naked-dsRNAs is their short-term stability. Some studies reported that nanoparticle-based delivery could enhance the stability and efficacy of exogenously applied dsRNAs when compared to naked-dsRNA delivery (Numata et al., 2014; Mitter et al., 2017). According to Numata et al. (2014), naked-dsRNA and ionic dsRNA-peptide complexes were infiltrated into *Arabidopsis thaliana* leaves to induce RNAi of the yellow fluorescent protein (*YEP*) transgene and chalcone synthase (*CHS*) endogenous gene. The peptide complex-based delivery of dsRNAs was found to downregulate the *YEP* expression within 12 h after infiltration, and the silencing effect was partly maintained for at least 36 h; whereas, the effect of naked-dsRNA on target genes was not significant. The dsRNA-peptide complexes were also found to be effective in downregulating the target *YFP* transgene in *Populus tremula* plant (Numata et al., 2014). In a subsequent study, the stability of exogenously applied naked-dsRNAs was compared with layered double hydroxide (LDH) clay nanosheets-based delivery (Mitter et al., 2017). Confocal microscopic analyses of Cy3 fluorophore labeled naked-dsRNA and LDH-loaded dsRNA targeting *CMV2b* found that most of the naked-dsRNA was washed away, whereas LDH-loaded dsRNA largely remained on the *A. thaliana* leaves. This study also reported that LDH-loaded dsRNA showed sustained release and were detectable after 30 days of application on sprayed *N. tabacum* leaves, but the naked-dsRNA was nearly undetectable after 20 days. Therefore, it was suggested that LDH nanosheets-based delivery can significantly improve the stability of exogenous dsRNA (Mitter et al., 2017). When combined with cationic fluorescent nanoparticles, dsRNAs also exhibited more dramatic suppression of target genes than naked-dsRNAs (Jiang et al., 2014). However, there are some reports where naked-dsRNAs proved effective. For instance, a study by Li et al. (2015) reported the naked-dsRNA induced suppression of target genes when exogenously applied to *Arabidopsis* and rice roots. In this study, naked-dsRNAs targeting MOB Kinase Activator 1A (*Mob1A*), and *Actin* genes were applied by root soaking in *Arabidopsis* and rice, respectively. In *Arabidopsis*, *Mob1A* regulates the root growth by controlling appropriate cell number and size and *Actin* plays a role as a cytoskeletal protein and also regulates root growth. The absorption of dsRNAs by *Arabidopsis* roots resulted in the suppression of root lengths and numbers as well as inhibited bolting and flowering. Whereas, *Actin* targeting dsRNAs absorbed by rice roots significantly suppressed root growth (Li et al., 2015). In another study, mechanical inoculation of naked-dsRNAs targeting the *MYB1* gene in the hybrid orchid plant was found to prominently reduce orchid flower buds (Lau et al., 2015). The *MYB* genes play an important role in the development of orchid varieties by regulating the pigmentation and morphogenesis of flowers.

Based on the above-mentioned studies, nanoparticle based-delivery techniques could facilitate the delivery of exogenous dsRNAs by increasing their stability and uptake (Numata et al., 2014; Jiang et al., 2014; Mitter et al., 2017). However, it is worth noting that nanotechnology is quite an expensive technology and also sensitive to the encapsulation process. Some other delivery methods like high-pressure spraying (Dalakouras et al., 2016) or direct exogenous application of naked-dsRNAs may also efficiently induce plant RNAi (Li et al., 2015; Lau et al., 2015; Dubrovina et al., 2019). Nevertheless, the efficiency of exogenously applied dsRNAs on plant RNAi are affected not only by application methods and delivery techniques but also the concentration and length of dsRNAs might play a crucial role (Tenllado and Diaz‐Ruiz, 2001; Carbonell et al., 2008; Dubrovina et al., 2019). Future studies in this direction could be beneficial for developing new technologies for the applications and delivery of dsRNAs towards inducing RNAi for the desired traits of the plants.

### Possible Uptake Mechanisms of Exogenous dsRNAs

Exogenously applied dsRNAs on the plant are absorbed into plant tissues and cells (Koch et al., 2016; Mitter et al., 2017; Dalakouras et al., 2018; Dubrovina and Kiselev, 2019), but it can be utilized to induce RNAi machinery in both plants and their invading pathogens (reviewed in Dubrovina and Kiselev, 2019; Wang and Dean, 2020). Therefore, the uptake of exogenously applied dsRNAs by plant cells is the most critical step. However, the uptake mechanism of exogenously applied dsRNAs is still elusive to much extent. One of the factors that could affect the efficiency of exogenous dsRNAs is the absorption capacity of different plant organs, e.g., leaves, petioles, buds, roots, stems, and seeds. According to Dalakouras et al. (2018), exogenously applied siRNAs by high-pressure spraying onto plant leaves and buds triggered local and systemic RNAi, whereas, delivery of siRNAs by petiole absorption and hpRNA by trunk injection failed to induce RNAi. Interestingly, application of 22-nt siRNA targeting *GFP* by bud spraying was more efficient to induce RNAi than leaf spraying in *N. benthamiana*. Whereas, siRNAs and hpRNA that were delivered by petiole absorption were present only in xylem tissues, but not in the apoplast. Based on these observations and others, the authors concluded that: (i) As a delivery method, high-pressure spraying is an efficient approach to deliver exogenous siRNAs into plant cells to induce RNAi; and (ii) As far as organ-based absorption is concerned, leaf and bud spraying efficiently induce RNAi compared to petiole absorption or trunk injection. The authors also hypothesized that the retention of exogenous dsRNAs/siRNAs on plant surfaces could be useful to deliver intact (unprocessed by plant) dsRNAs. These intact dsRNAs if taken up by target insects/fungi, could result in pest and disease management (Dalakouras et al., 2018). In another study, Koch et al. (2016) analyzed the detection of spray-applied dsRNAs labeled with the green fluorescent dye (ATTO 488) in barley leaf. Using confocal laser microscopy, the authors detected the green fluorescent signal from fluorescing dsRNAs in the xylem, apoplast, symplast of phloem parenchyma cells, companion cells, and mesophyll cells, along with trichomes and stomata. Furthermore, topically applied naked- and bio-clay loaded dsRNAs labeled with Cy3 were observed in the xylem of *Arabidopsis* leaves (Mitter et al., 2017), but only bio-clay loaded dsRNAs showed larger uptake into the spongy mesophyll. Song et al. (2018) investigated the cellular uptake of spray-applied dsRNAs into wheat cells using healthy and wounded coleoptiles. The results indicated that dsRNA uptake was more efficient through the wounded surface than the healthy surface. Based on microscopic analyses, it was hypothesized that exogenous dsRNAs transferred *via* tracheary elements after entering into the damaged cells of the wounded coleoptiles.

The exact mechanisms underlying perception, recognition, and translocation of exogenously applied dsRNAs into the plant cell are still unknown. Plant-microbe interactions mediated extracellular DNA (eDNA) or RNA (eRNA) perception by plants crucially regulates self- and non-self-recognition and induces pattern-triggered immunity (PTI) (Niehl et al., 2016; Bhat and Ryu, 2016). It is proposed that the biomolecular markers of microbe- and pathogen-associated molecular patterns (MAMPs and PAMPs) in plants are perceived by cell-surface proteins called pattern-recognition receptors (PRRs). Upon recognition, signal transduction cascades are triggered by PRRs to induce the plant's innate immunity system, also called PTI (Niehl et al., 2016; Bhat and Ryu, 2016). Indeed, RNAs served as MAMPs and induced PTI responses when exogenously applied to *Arabidopsis* (Lee et al., 2016). According to this study, *Arabidopsis* leaves pre-infiltrated with total RNAs purified from *Pseudomonas syringae* pv. tomato DC3000 (*Pto* DC3000), elicited plant immune responses similar to those induced against *Pto* DC3000 bacterium, suggesting that total bacterial RNAs could trigger plant innate immunity responses. Yakushiji et al. (2009) investigated the elicitor activity of bacterial DNA in *Arabidopsis* and the results indicated that non-methylated CpG DNAs served as MAMPs and induced defense responses. Although the mechanisms underlying the recognition and cellular uptake of extracellular DNA by receptors have not been identified in plants, a study reported by Niehl et al. (2016) showed that purified dsRNAs from virus-infected plants and synthetic dsRNA analogs, both induced PTI responses in *Arabidopsis*. Exogenous dsRNA-induced PTI responses were dependent on the co-receptor SOMATIC EMBRYOGENESIS RECEPTOR KINASE 1 (*SERK1*) but were DCL- independent. Thus, it was proposed that membrane-bound *SERK1*could act as a potential dsRNA receptor. In this regard, global transcriptomics and proteomics analysis may help to screen trans-membrane marker proteins and genes for elucidating the receptor-mediated recognition, perception, and uptake of dsRNAs into plant cells.

## The Possible Fate of dsRNAs Into the Plant Cell

### Gene Silencing, Transitivity, and DNA Methylation

**Figure 2:**
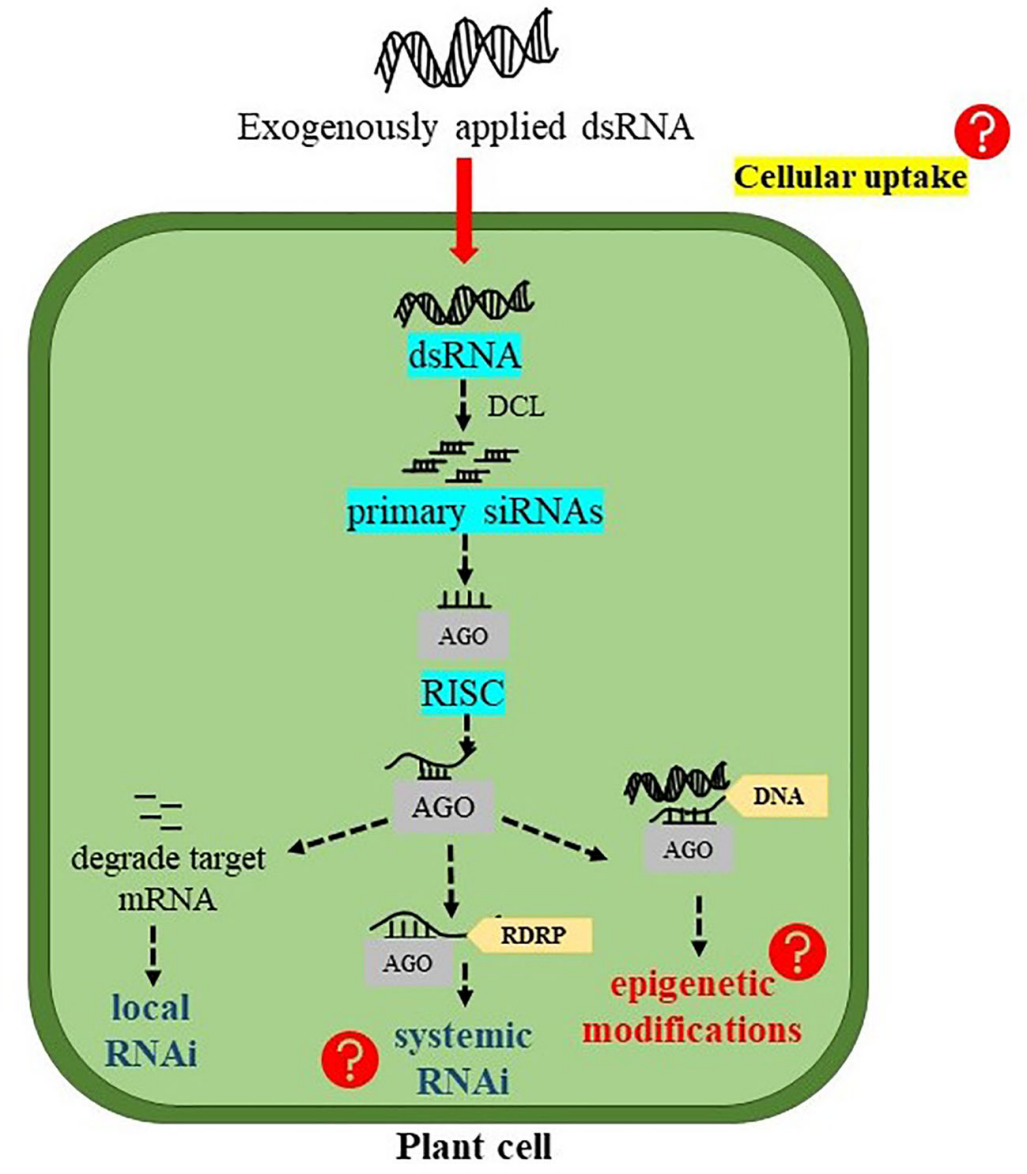
Possible fate of exogenously applied double-stranded RNA (dsRNA) into plant cells. DCL, DICER-LIKE endonucleases; siRNAs, short interfering RNAs; AGO, ARGONAUTE protein; RISC, RNA-induced silencing complex; and RDRP, RNA DEPENDENT RNA POLYMERASE.

The principal role of dsRNAs is to trigger RNAi machinery in the plant system which involves several processing steps (**Figure 2**). In plant cells, dsRNAs are first processed into small nucleotide primary siRNAs by DCL endonuclease. Four paralogues of DCL including DCL1, DCL2, DCL3, and DCL4 have been found in *A. thaliana* (Bologna and Voinnet, 2014). Among them, DCL2, DCL3, and DCL4 process dsRNAs into 22-, 24-, and 21- nucleotide siRNAs, respectively. These primary siRNAs are then incorporated onto AGO proteins to form RISC. Generally, the 21-nt siRNAs are loaded on AGO1, and target complementary mRNA transcripts for cleavage and degradation, resulting in “PTGS” or RNAi (Dalakouras et al., 2020). In *Arabidopsis,* among 10 identified AGO genes, AGO1 mainly found to initiate PTGS whereas, AGO4 mediates transcriptional gene silencing (TGS) (Bologna and Voinnet, 2014). On the other hand, when 22-nt siRNAs are loaded on AGO1, they recruit RNA DEPENDENT RNA POLYMERASE 6 (RDR6) to the target RNA transcript and transcribing it to dsRNAs. These newly synthesized dsRNAs lead to the generation of secondary siRNAs and the amplification of silencing signals, in a mechanism coined “transitive” silencing (Chen et al., 2010; Cuperus et al., 2010). A study conducted by Dalakouras et al. (2016) investigated the effects of size and structure of exogenous sRNAs (21-, 22-, and 24-nt sRNAs either as a perfect duplex or as sRNAs containing an asymmetric bulge) on local, transitive, and systemic RNAi in *GFP*-expressing *N. benthamiana* plant. The results indicated that all sRNAs were able to initiate local RNAi, whereas, only 22-nt sRNAs, in both forms, were able to induce systemic RNAi. DCL2 is responsible for processing dsRNAs into 22-nt siRNAs (Henderson et al., 2006), which then recruit RDR6 to initiate transitivity (Chen et al., 2010; Cuperus et al., 2010). In a recent study, transcript levels of *NPTII* and *EGFP* transgenes were significantly reduced after treating the transgenic *Arabidopsis* plants with the corresponding dsRNAs of these genes. The induction of the RNAi machinery in treated plants has become evident after the detection of EGFP-derived siRNA in the treated plants. However, what was surprising is that EGFP-derived siRNA was also detected in the plants treated with NPTII-dsRNA indicating that *NPTII* silencing could also be mediated by silencing transitivity. Indeed, the analysis of read-through transcripts of both *NPTII* and *EGFP* in treated and untreated transgenic *Arabidopsis* indicated that silencing transitivity has probably taken place even before treatments by dsRNAs, but was enhanced after dsRNA treatments. Supporting this assumption is the findings that *NPTII* transcript levels were downregulated in plants treated with EGFP-dsRNA and vice versa (Dubrovina and Kiselev, 2019).

DsRNAs can also be processed by DCL3 to produce 24-nt siRNAs. When 24-nt siRNAs are loaded on AGO4, they recognize cognate DNAs and recruit Pol V (a DNA dependent RNA polymerase) to form “RNA-directed DNA methylation” (RdDM) (Chen et al., 2010). Actually, RdDM is necessary for the *de novo* cytosine methylation primarily within the region of RNA-DNA sequence identity. In plants, DNA methylation can occur in all three sequence contexts (symmetric CG, CHG, and asymmetric CHH), and the RdDM pathway was found to methylate all sequence contexts (Singroha and Sharma, 2019). DsRNA induced RNAi machinery stimulates not only RdRM but also histone modifications which play an important role in epigenetic transcriptional gene silencing (Wassenegger, 2005). According to the study conducted by Dubrovina and Kiselev (2019), the exogenously applied dsRNAs targeting *EGFP* and *NPTII* in transgenic *Arabidopsis* considerably increased the cytosine DNA methylation at three different contexts of CG, CHG, and CHH. This study demonstrated that DNA methylation at the coding sequences of the *EGFP* and *NPTII* transgene could reflect the influence of 24-nt siRNAs. There are possibilities that exo-dsRNAs treatment initiates inductions and spreading of DNA methylation at the T-DNA regions bearing both *EGFP* and *NPTII* transgene, which could promote the transgene mRNA degradation as well as affect transcription of the transgene and/or heterochromatin formation (Dubrovina and Kiselev, 2019).

### Short-Distance and Long-Distance Cellular Movement of sRNAs

sRNAs can move short and long-distance throughout the plant cells (Sarkies and Miska, 2014). Primary siRNAs are able to spread for short-range (10–15 cells), through the symplastic route, without producing secondary siRNAs (Kim, 2005). Symplastic movements of sRNAs require the passage through the plasmodesmata. The size exclusion limit of plasmodesmata is around 30 to 50 kDa which should not limit the passing of naked sRNAs, but it might affect the transport of sRNAs that are enclosed in vesicles and/or bound to RNA binding proteins (Wang and Dean, 2020). During the development of plant organs, plasmodesmata can change their size and selectivity that may allow the passage of sRNAs (Imlau et al., 1999). Long-distance movement or systemic silencing has been found to be phloem-mediated that requires amplification of the silencing signals by RDRPs (Chen et al., 2010; Cuperus et al., 2010; Liang et al., 2012; Dalakouras et al., 2018). The generation of secondary siRNAs is led by transitive silencing cascades and amplification of silencing. In *Arabidopsis*, DCL2 is known to play an essential role in the accumulation of secondary siRNAs and silencing transitivity (Chen et al., 2010; Cuperus et al., 2010). In vascular plants, phloem serves as a highway for mobile signals. Several lines of evidence indicated that the symplastic signaling follows the photoassimilate translocation route from source to sink tissues, which involves reaching the companion cells (specialized cells of the phloem tissue) through plasmodesmata. Then signal molecules could transfer from here to the other phloem‐specific types of cells called sieve elements. The sieve elements are known as end‐to‐end connected enlarged cells which create a channel for fast communication to distant organs (Mermigka et al., 2016). The phloem-based source to sink movement of the systemic sRNAs signal is also reported by some studies (Pant et al., 2008; Liang et al., 2012; Zhang et al., 2014; Patil and Fauquet, 2015). Different types of RNAs have been found in the phloem exudate including viral RNAs, siRNAs, miRNAs, transfer RNAs, and messenger RNAs (Kehr and Buhtz, 2008). For sRNAs movement throughout the plants, RNA binding proteins play important roles (Wang and Dean, 2020). However, the exact mechanism underlying the functions of sRNAs derived from exogenous dsRNAs in mobile signaling is still largely unknown.

### Exogenous dsRNAs Induced RNAi in Plant-Microbe Interactions

Exogenous applications of dsRNAs have been reported to induce plant resistance against pathogens by activating the RNAi machinery (Tenllado and Diaz‐Ruiz, 2001; Numata et al., 2014; Kamthan et al., 2015; Wang et al., 2016; Guo et al., 2016; Mitter et al., 2017; Dubrovina and Kiselev, 2019; Dalakouras et al., 2020). In this section, inhibitory activities of exogenous dsRNAs on fungi and viruses are summarized.

### RNAi-Mediated Resistance Against Phytopathogenic Fungi

To control fungal diseases in the plant, spray-induced gene silencing (SIGS) is currently considered as an innovative, eco-friendly, biological tool that involves an exogenous spray of dsRNAs or siRNAs onto plant surface. Exogenously applied dsRNAs and siRNAs have been reported to protect several plant species including barley, tomato, strawberry, grape, oilseed rape, wheat, onion, rose, lettuce, cucumber, soybean, and *Arabidopsis* against several fungi such as *Fusarium graminearum*, *Botrytis cinerea*, *Sclerotinia sclerotiorum,* and *Fusarium asiaticum* (Koch et al., 2016; Wang et al., 2016; McLoughlin et al., 2018; Song et al., 2018; Gu et al., 2019).

According to Koch et al. (2016), spray applications of dsRNAs and siRNAs onto barley detached leaves attenuated fungal diseases by inhibiting fungal growth and suppressing three fungal cytochrome P450 genes, i.e., *CYP51A*, *CYP51B*, *CYP51C*, of *F. graminearum*. In another study, foliar applications of dsRNAs onto oilseed rape and *Arabidopsis* leaf surface exhibited antifungal potential against *S. sclerotiorum* and *B. cinerea* (McLoughlin et al., 2018). A recent study conducted by Song et al. (2018) also found that spraying of dsRNAs targeting the *Myo5* gene of *F. asiaticum* resulted in reduced fungal virulence in wheat. However, the RNAi effect was maintained only when the continuous supply of dsRNA was provided. Based on sRNA deep sequencing analysis, it was revealed that *F*. *asiaticum* was not able to amplify secondary siRNAs, whereas, *Myo5*-dsRNA derived siRNAs were detected in plant cells. These results indicate that in fungi, RNAi is not maintained by RDRP amplification loop (Song et al., 2018). Exogenous applications of dsRNAs and siRNAs targeting *DCL1* and *DCL2* genes of *B*. *cinerea* was found to reduce fungal virulence in fruits (e.g., tomato, strawberry, and grape), vegetables (e.g., lettuce and onion), and flower petals (e.g., rose) (Wang et al., 2016). Exogenously applied dsRNAs and siRNAs can induce resistance against fungi, either indirectly through their uptake by host plant cells and then their introduction to fungal cells, or directly through fungal cells that uptake dsRNA/siRNA, leading to silencing of target genes (Koch et al., 2016; Wang et al., 2016; Song et al., 2018). Based on these abovementioned findings, it is reasonable to conclude that dsRNAs/siRNAs could be used as biofungicides to control phytopathogenic fungi, pre- and post-harvest. However, more investigations are still needed to find out suitable/stable application methods, delivery techniques, effective time periods, uptake regulatory factors, and mechanisms underlying the translocation of dsRNAs/siRNAs between plant and fungal cells.

### RNAi-Mediated Resistance Against Viruses

Several studies reported that foliar applications of dsRNAs induce plant resistance against target viruses (Tenllado and Diaz‐Ruiz, 2001; Carbonell et al., 2008; Konakalla et al., 2016; Kaldis et al., 2018; Worrall et al., 2019; Vadlamudi et al., 2020). The effects of exogenously applied dsRNAs on conferring resistance against viruses have been reported in various host species, including tomato, tobacco, maize, papaya, cowpea, cucumber, watermelon, and squash against different viruses such as tobacco etch virus (TEV), tobacco mosaic virus (TMV), alfalfa mosaic virus (AMV), pepper mild mottle virus (PMMoV), potyvirus, bean common mosaic virus (BCMV), papaya ringspot virus (PRSV), and zucchini yellow mosaic virus (ZYMV) (reviewed in Dubrovina and Kiselev, 2019). These studies demonstrated that dsRNA-treated plants are capable of triggering RNAi-mediated processes to reduce or delay viral infection by silencing target viral genes.

Exogenously applied dsRNAs on plants for antiviral effects were first reported by Tenllado and Diaz‐Ruiz (2001). According to this study, dsRNAs targeting the replicase protein (*RP*) gene of PMMoV, TEV, and AMV attenuated viral infections when introduced in tobacco leaves along with the target virus. Other studies demonstrated that exogenously applied dsRNAs were effective in conferring protection against TMV in tobacco (Yin et al., 2009; Konakalla et al., 2016; Niehl et al., 2018); PMMoV in tobacco (Tenllado et al., +2003); ZYMV in cucumber, watermelon, and squash plants (Kaldis et al., 2018). A recent study conducted by Vadlamudi et al. (2020) demonstrated that the topical application of dsRNAs molecules derived from both *CP* and *HC-Pro* genes of the PRSV-Tirupati isolate conferred resistance to papaya plants against PRSV-Tirupati and PRSV-Delhi viral isolates. The results found that the dsRNA molecules conferred 100% resistance against PRSV-Tirupati infection and the same dsRNA molecules were highly effective against the PRSV-Delhi isolate conferring resistance of 94% and 81%, respectively. The great concern regarding the possible instability of naked-dsRNA applications could account for the short-term protection against viral pathogens. To address such a concern, a recent study demonstrated that a single application of LDH-loaded dsRNAs effectively provided RNAi-mediated virus protection and its effect lasted for at least 20 days in cowpea leaves (Mitter et al., 2017). According to this study, the exogenous spray of LDH-loaded dsRNAs in tobacco and cowpea was found to induce resistance against PMMoV and CMV by targeting *RP* and *2b suppressor* genes, respectively (Mitter et al., 2017). In a subsequent study, spraying of LDH-loaded dsRNA on tobacco and cowpea was also found to provide resistance to BCMV infections (Worrall et al., 2019). Unfortunately, the efficacy of exogenously applied dsRNAs against viral pathogenes have largely been investigated under controlled experimental conditions. Therefore, it is still unclear if pretreating the plants with dsRNAs to prevent the subsequent viral infection or using dsRNAs to treat the existing viral infection could be commercially feasible under open field conditions.

## Potential Applications of Exogenous dsRNAs in Crop Improvement

The RNAi technology that is based on transgenic plant generation has been widely applied in crop improvement, development, and disease management by manipulating the expression of target genes. For plant protection, RNAi-based genetic transformation, also refers to as host-induced-gene silencing (HIGS), allows for the silencing of target genes in plant pathogens, by expressing RNAi constructs in the host plant (Qi et al., 2019). RNAi-based transgenic plants have also been developed for crop improvement, plant development, and other desired traits, by expressing RNAi constructs in plants to suppress target genes. The roles of RNAi in crop improvement have been demonstrated in the development of seedless fruits, plant biomass regulation, flower coloration, scent development, shelf-life enhancement, secondary metabolite regulation, and abiotic stress tolerance (Saurabh et al., 2014; Kamthan et al., 2015; Guo et al., 2016). Despite the importance of this technology and its implementations in modern agricultural systems, RNAi applications *via* permanent genetic transformation have raised public concerns regarding their long-term consequences on ecological stability and human health. This, along with the technical challenges that might face its applications on several crops paves the way for exogenous dsRNA-induced RNAi as another alternative that is generally perceived as minimally invasive, efficient, target-specific, eco-friendly, and capable of being applied to various crops regardless of their genetic backgrounds (Jiang et al., 2014; Li et al., 2015; Lau et al., 2015). A few examples of RNAi technology-based on transgenic plant generation in crop improvement and development were discussed below, to draw an image of how exogenously applied dsRNAs could be applied in the future to replace permanent genetic transformation technologies.

The shelf life of fruits and vegetables is a crucial factor responsible for post-harvest deterioration and spoilage, which results in major economic losses. The RNAi technologies have been applied to increase the shelf life of fruits and vegetables by delaying ripening. Generally, climacteric fruit ripening is initiated by ethylene, a plant growth regulator that regulates ripening-related genes and pathways (Osorio et al., 2011). The RNAi technology was applied to generate transgenic tomato plants by introducing a dsRNA unit targeting *1-Aminocyclopropane-1-carboxylate* (*ACC*) oxidase gene, which catalyzes the oxidation of *ACC* to ethylene (Xiong et al., 2005). Transgenic tomatoes with impaired *ACC* released only trace amounts of ethylene and had a shelf-life of more than 120 days. Other fruit ripening-related targets include *α-mannosidase* (*α-Man*) and *β-* d *-N-acetylhexosaminidase* (*β-Hex*). RNAi technology was used to generate the transgenic tomato plant by introducing hpRNAs targeting *α-Man* and *β-Hex* (Meli et al., 2010). The results indicated that RNAi suppression of both *α-Man* and *β-Hex* genes reduced softness and therefore extended the shelf life of tomatoes for nearly 30 days.

RNAi-based genetic transformation was also applied to control branch development and increase the total number of branches in kiwi by targeting the carotenoid cleavage dioxygenase (*CCD*) gene (Ledger et al., 2010). Flower color regulation was also achieved using RNAi technology (Fukusaki et al., 2004; Nishihara et al., 2005). According to Fukusaki et al. (2004), the original blue flower color of *Torenia hybrida* was modulated to exhibit white and pale color by employing RNAi technology targeting *CHS*. RNAi was also used to generate parthenocarpic (seedless) tomatoes by targeting *CHS* to down-regulate the flavonoid biosynthesis pathway (Schijlen et al., 2007), or manipulating the biosynthesis or signaling of phytohormones such as auxin and gibberellins (De Jong et al., 2009).

Plant primary and secondary metabolites including phenolics, flavonoids, phenolic acids, amino acids, etc. not only play important roles in maintaining the physicochemical properties of the plant but also possesses numerous human health benefits (Das and Eun, 2016; Das and Eun, 2018; Das et al., 2019). RNAi applications to regulate plant metabolite profiling have been contributed to the nutritional improvement, biofortification, and allergen or toxin elimination (Saurabh et al., 2014; Guo et al., 2016). The RNAi technology was applied to enhance the carotenoid and flavonoid production in tomato (Davuluri et al., 2005). In another study, transgenic *Artemisia annua* plants were generated using the hpRNA‐mediated RNAi technique targeting *squalene synthase* (*SQS*) (Zhang et al., 2009). The suppression of *SQS*, a key enzyme in the sterol pathway significantly increased the artemisinin content in transgenic plants. In conclusion, all these reports indicate how RNAi technology could be applied to positively affect several aspects of plant growth, development, ripening, nutritional content, and physiology. However, whether exogenous dsRNA-induced RNAi could efficiently mediate these roles, is still largely unknown due to the lack of research in this area.

## Production Technologies and Cost of dsRNAs

Generally, *in vitro* and *in vivo* methods that utilize the DNA dependent RNA polymerase (DdRP) from bacteriophage T7 for transcription of target-specific sequences are used for dsRNA production (Voloudakis et al., 2015). The production of dsRNAs using *in vitro* transcription systems requires the use of commercial systems (kit). So far, the commercial kits used for dsRNAs production are quite expensive, limited to small-scale production, and prone to false amplification, which may lead to poor quality of dsRNA products. The production of dsRNAs using *in vivo* methods involves the use of bacteria (e.g. *Escherichia coli* and *Pseudomonas syringae*) and yeast (*Yarrowia lipolytica*) (Voloudakis et al., 2015; Alvarez-Sanchez et al., 2017). “RNAgri” agricultural industry developed microbial fermentation technology to manufacture dsRNAs at a larger-scale. This industry utilizes a protein to bind the desired RNAs, hence protecting them against degradation. The final dsRNA products are considered safe to use and stable than naked dsRNAs (http://www.rnagri.com/). In comparison to the *in vitro* transcription system, microbial-based dsRNAs production by prokaryotic or eukaryotic cells is considered as a sustainable strategy for providing large quantities of dsRNAs (Voloudakis et al., 2015). The increasing demand for dsRNAs requires a production system, that is scalable and cost-efficient. It is assumed that approximately 2 to 10 g of dsRNAs are required per each hectare of arable land, and this may even vary based on the target species' sensitivity to RNAi, systemic silencing capacity, and application method as well as delivery techniques. The *in vitro* dsRNAs production cost using nucleoside triphosphate (NPT) synthesis was nearly $12,500/gm in 2008, but then decreased to $100 in 2016, and $60 today (Andrade and Hunter, 2016; Zotti et al., 2018; Dalakouras et al., 2020). More recently, to meet the high market demand, several industrial companies are now shifting to microbial-based production systems to manufacture dsRNAs at a larg-scale and nearly at 2 USD/gm (Zotti et al., 2018; Dalakouras et al., 2020). Using bacterial minicells is another promising technology that is currently utilized for the production and encapsulation of dsRNAs. If successful, this technology could provide better shielding and slow and sustained release of dsRNAs for agricultural purposes under open-field conditions (http://www.agrospheres.com).

## Concluding Remarks

RNA interference (RNAi) technology using the exogenous application of double-stranded RNAs (dsRNAs), short interfering RNAs (siRNAs), and hairpin RNAs (hpRNAs) have emerged as a potential tool for improving various agronomically important plants. However, several critical factors need to be clarified for proper, effective, and safe utilization of these tools as sustainable solutions for modern crop protection and improvement. Optimization of the concentration and length of dsRNAs is a very critical factor for effective RNAi. To induce effective silencing, dsRNAs length and dose have to be determined for individual target genes and plant species. The suitable application method and delivery technique are also highly important because it may critically affect the cellular absorption rate of exogenous dsRNAs and their stability under open-field conditions. Along the same line, the elucidation of the cellular uptake mechanisms of exogenous dsRNAs in plants and their invading organisms is of great importance. In cases of trans-membrane receptor-mediated cellular uptake, advanced proteomics and transcriptomics analyses, as well as functional genomic approaches could provide some important insights in that regard. Furthermore, the mechanisms underlying the siRNAs (derived from exogenous dsRNAs) movement throughout the plant cells as well as the cellular processing of exogenously applied dsRNAs and their proportional involvements in post-transcriptional gene silencing, systemic silencing, and epigenetic modifications of target genes remains to be elucidated. Another line of research that also requires more attention is whether exogenous dsRNA-based approaches could provide a feasible alternative to genetic transformation methods, especially with regards to manipulating endogenous genes to modulate plant growth and development. Also, the applications of dsRNAs for spray-induced gene silencing have already demonstrated great success in disease management at least at the research level. Nevertheless, the uptake mechanisms of dsRNAs in both host plants and their invading organisms are not completely understood. Elucidating these mechanisms is not only important at the scientific levels, but it could also lead to exploring new dsRNA delivery and encapsulation techniques as well as optimizing dsRNA concentrations and lengths to ensure better stability and long-lasting efficacy. In conclusion, it is reasonable to assume that, exogenous dsRNA induced RNAi technology could be the gate for more eco-friendly and sustainable practices for the regulation of genes related to disease management, plant development, and crop improvement. However, addressing the above-mentioned queries and others pertinent to production technologies and cost is substantial in order to move this technology from research to large-scale agricultural applications in greenhouses and open-fields.

## Author Contributions

PD and SS have contributed to the writing, editing, and preparation of this review article.

## Funding

This work was supported by The Virginia Catalyst (fund# 460380) and The Institute for Critical Technology and Applied Science (ICTAS) (fund# 178811).

## Conflict of Interest

The authors declare that the research was conducted in the absence of any commercial or financial relationships that could be construed as a potential conflict of interest.
